# Apoptosis of cancer cells is triggered by selective crosslinking and inhibition of receptor tyrosine kinases

**DOI:** 10.1038/s42003-019-0484-5

**Published:** 2019-06-21

**Authors:** Kaidi Wang, Xuan Wang, Yiying Hou, Huihui Zhou, Kangsen Mai, Gen He

**Affiliations:** 1 Key Laboratory of Mariculture, Ministry of Education, Ocean University of China, 266003 Qingdao, China; 2 Laboratory for Marine Fisheries Science and Food Production Processes, Qingdao National Laboratory for Marine Science and Technology, 266003 Qingdao, China

**Keywords:** Apoptosis, Target identification, Lectins

## Abstract

Receptor tyrosine kinases (RTK) have been the most prevalent therapeutic targets in anti-cancer drug development. However, the emergence of drug resistance toward single target RTK inhibitors remains a major challenge to achieve long-term remissions. Development of alternative RTK inhibitory strategies that bypass drug resistance is much wanted. In the present study, we found that selected cell surface RTKs were inhibited and crosslinked into detergent resistant complexes by oligomeric but not monomeric concanavalin A (ConA). The inhibition of RTKs by ConA led to suppression of pro-survival pathways and induction of apoptosis in multiple cancer cell lines, while overexpression of constitutively activated protein kinase B (AKT) reversed the apoptotic effect. However, major cell stress sensing checkpoints were not influenced by ConA. To our knowledge, selective crosslinking and inhibition of cell surface receptors by ConA-like molecules might represent a previously unidentified mechanism that could be potentially exploited for therapeutic development.

## Abstract (web summary)

Kaidi Wang et al. show that the inhibition of receptor tyrosine kinases by concanavalin A suppresses pro-survival pathways, triggering apoptosis. This study suggests that selective crosslinking of receptor tyrosine kinases with concanavalin A -like molecules may be exploited as an anti-cancer strategy.

## Introduction

Targeted cancer therapies have received substantial successes in clinics and been the focus of drug development^1^. Receptor tyrosine kinases (RTKs) are the major targets for such approaches, because of their critical roles in cell survival and proliferation, and aberrantly activated in a wide range of cancers^2^. However, cancer cells can become resistant to single target RTK inhibitors. This occurs either through mutations at the gatekeeper residues of ATP binding pocket that disrupt the interactions of RTK inhibitor with the kinase^3^, or bypassing mechanisms involving amplification of an alternative RTK that is not primarily targeted^4^.

Multiple approaches have been made to overcome drug resistance under different circumstances. Allosteric inhibitors that target different region of the kinase were proposed to avoid mutant-related resistances^5^. Compounds and methods that selectively degrade oncogenic kinase targets were also reported^6^. Multiple target therapies were developed either by combination of single RTK inhibitors or administration of a single compound targeting multiple RTKs^7,8^. Nevertheless, these strategies are still limited by the increased toxicity associated with indiscriminative signaling inhibition in normal cells^9^.

Alternatively, efforts have been taken to focus on cellular processes that cancers exploit and disproportionately rely on^10^. Cumulative evidence shows that cancer cells exhibit a completely different repertoire of glycan structures compared with their normal counterparts^11^. The most-widely occurring cancer-associated changes in glycosylation are sialylation, fucosylation, O-glycan truncation, and N- and O-linked glycan branching^12^. Differential glycosylation has been found to participate in multiple processes of cancer, including inflammation, immune surveillance, cell adhesion, intra-and inter-cellular signaling and metabolism^11^. Notably, changes in the pattern of glycosylation of cell surface receptors also influence the sensitivity of target therapy in cancer cells and impact the acquisition of drug resistance^13^. Targeting altered glycosylation has thus been considered a new and relatively unexploited strategy for drug development^14^.

Several therapeutic approaches have been made to target glycosylation. Immunization with carbohydrate antigens for a potential vaccination in cancer immunotherapy was explored but hampered by poor immunological response induced by such glycans^15^. Inhibitors are developed against galectins that are carbohydrate-binding proteins actively involved in promoting cancer progression and metastasis^16^, but still remain to be tested in clinical trials. Glycol-biosynthesis machinery also represents a potential point of intervention. However, it still faces enormous challenge to discriminate between cancer and normal cells in such approach^17^.

In the present study, we report a mechanism of multiple RTK inhibition through targeting their carbohydrate moieties by concanavalin A (ConA), which induces apoptosis and potentially discriminate between cancer and normal cells^18^. This strategy should provide potential to bypass drug resistance associated with single target RTK inhibitors, as well as toxicity of multiple RTK inhibitions caused by indiscriminative targeting toward normal cells in previous approaches.

## Results

### ConA-induced apoptosis in cancerous cell lines

**Fig. 1:**
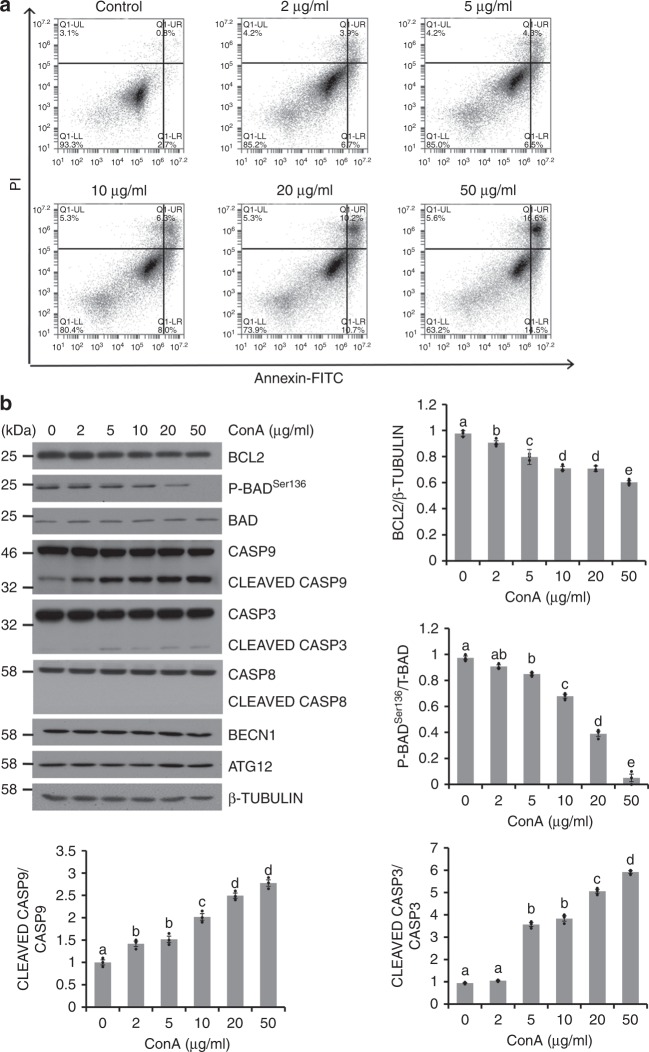
ConA induced cell apoptosis. **a** Hela cells were treated with ConA for 9 h and stained with Annexin V-FITC/PI for apoptosis analyses using flow cytometry. **b** Cell lysates were collected after incubation with ConA for 9 h. The levels and activities of proteins involved in apoptosis and autophagy were examined by western blot and quantitated. Results were represented as means with standard errors (*n* = 3) and analyzed using one-way ANOVA. Values with different letters in the same column (a–e) were significantly different (*p* < 0.05) from each other

Along with previous studies conducted in other cell lines^18^, the apoptotic effect of ConA was further examined in human cervical (Hela), colorectal (Caco-2), and lung (A549) carcinoma cells. Quantitated by Annexin V-FITC/propidium iodide (PI) staining, the apoptotic ratio was reached from 3.6 ± 0.7–31.2 ± 1.5% in a dose dependent manner after Hela cells were treated with serial concentrations of ConA at 0, 2, 5, 10, 20, 50 μg/ml for 9 h (Fig. 1a, Supplementary Fig. 1a). For the key molecules involved in apoptosis initiation, both the phosphorylation levels of anti-apoptotic B-cell lymphoma-2 (BCL2) and BCL2-associated death promoter (BAD) were reduced after ConA treatment (Fig. 1b). Furthermore, ConA stimulated the cleavage/activation of caspase (CASP) 3 and 9, but not that of CASP8, in the execution phase of cell apoptosis. These results were further confirmed in Caco-2 and A549 cell lines (Supplementary Fig. 1b). However, the protein levels of cellular autophagy markers, both BECN1 and autophagy related 12 (ATG12), were not influenced by ConA under the present experimental conditions (Fig. 1b).

### The effect of ConA on activation of cell surface receptors

**Fig. 2:**
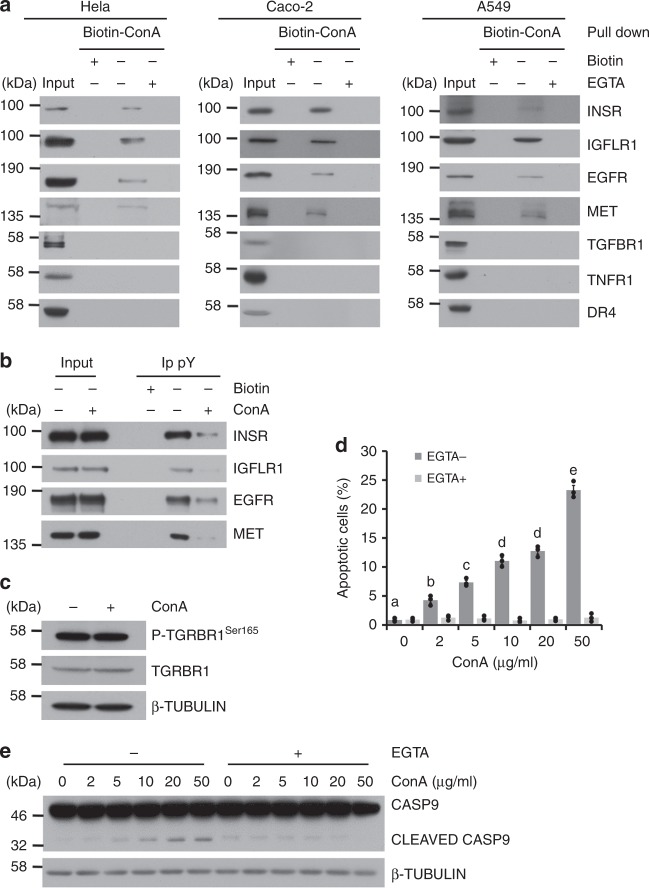
The effect of ConA on activation of cell surface receptors. **a** Cell surface receptors were pulled down using biotinylated ConA with or without 5 mM EGTA in different cell lines. **b** Hela Cells were treated with ConA for 4 h and stimulated with full medium for 15 mins. The phosphorylation of cell surface RTKs was examined by immunoprecipitation using a phosphotyrosine (pY) antibody. **c** As a control, the phosphorylation and total levels of TGFBR1 were determined by direct western blot. **d** Annexin V-FITC/PI analysis was done after EGTA and ConA treatment. **e** Hela cells were co-incubated with 5 mM EGTA and ConA at indicated concentrations for 9 h. The levels of Pro/cleaved CASP9 were examined by western blot. Results were represented as means with standard errors (*n* = 3) and analyzed using one-way ANOVA. Values with different letters (**a**–**e**) in the same column were significantly different (*p* < 0.05) from each other

The interactions between ConA and cell surface receptors were examined by affinity chromatography using biotinylated ConA. Receptors including insulin receptor (INSR), IGF-1 receptor (IGFLR1), EGF receptor (EGFR), and hepatocyte growth factor receptor (MET) were pulled down by biotinylated ConA. However, transforming growth factor β receptor (TGFBR1), tumor necrosis factor receptor 1 (TNFR1), and death receptor 4 (DR4) showed no apparent interaction with ConA. These results were demonstrated in Hela, Caco-2, and A549 cells (Fig. 2a). The activities of these cell surface receptors were further examined by immunoprecipitation using a phosphotyrosine antibody. As shown in Fig. 2b, the phosphorylated forms of INSR, IGFLR1, EGFR, and MET were reduced after cells were treated with ConA at 10 μg/ml for 4 h. However, the phosphorylation of TGFBR1 was not influenced (Fig. 2c). In order to correlate the receptor binding and inhibition by ConA with its apoptotic effects, we treated Hela cells with ConA in the presence of EGTA, which was known to chelate ions and abolish the ConA binding to glycoproteins^19^ (Fig. 2a). As shown in Fig. 2d, e, EGTA fully abolished ConA’s effect on cell apoptosis, judged by both Annexin V-PI staining (Fig. 2d, Supplementary Fig. 2a) and cleavages of CASP9 (Fig. 2e).

### The influences of ConA on intracellular signaling

**Fig. 3:**
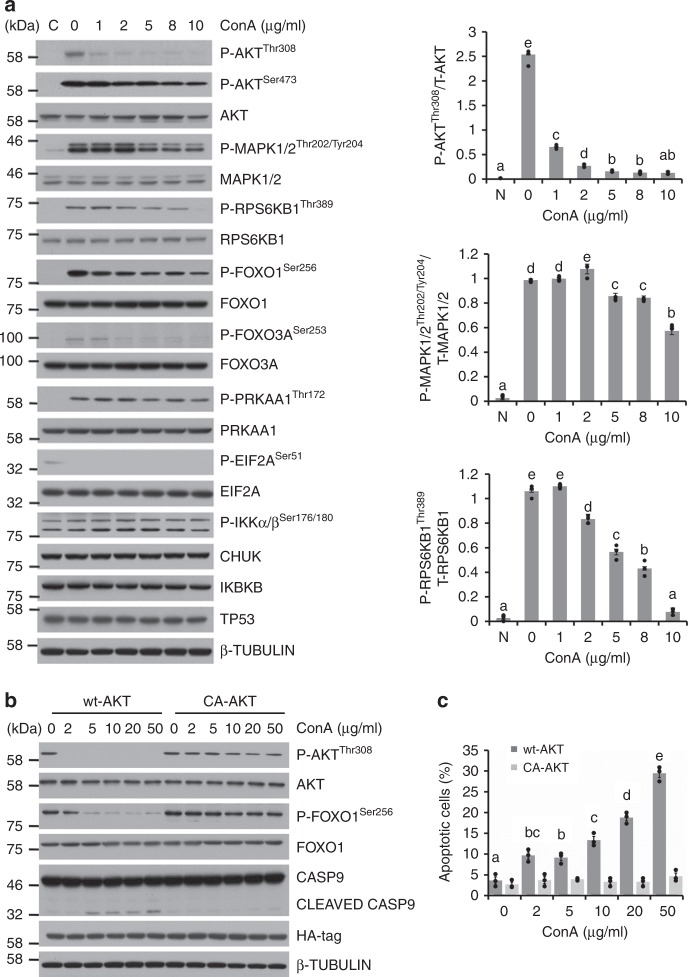
The influences of ConA on intracellular signaling. **a** Hela cells were treated with ConA for 4 h and stimulated with full medium for 15 mins. The levels and activities of key signaling molecules involved in survival and stress pathways were examined by western blot and quantitated. **b** The levels and activities of proteins involved in apoptosis in AKT overexpressing cells were examined by western blots and quantitated. **c** Hela cells overexpressing wild type (wt-AKT) or constitutively activated AKT (CA-AKT) were treated with ConA for 9 h and stained with Annexin V-FITC/PI for apoptosis analysis. Results were represented as means with standard errors (*n* = 3) and analyzed using one-way ANOVA. Values with different letters in the same column (**a**–**e**) were significantly different (*p* < 0.05) from each other

To further evaluate the cellular effects of ConA, the activities of multiple key intracellular signaling molecules were examined. As shown in Fig. 3a, after 4 h of treatment at serial concentrations in Hela cells, ConA suppressed the major pro-survival pathways that were downstream of cell surface RTKs. These include phosphatidyl-inositol 3-kinase (PI3K), extracellular signal-regulated protein kinases 1 and 2 (MAPK/ERK), mammalian target of rapamycin (mTOR) pathways, characterized by the reduced phosphorylation levels of AKT, MAPK1/2, and p70 S6 Kinase (RPS6KB1). Most notably, AKT phosphorylation was markedly reduced by ConA treatment. In addition, ConA suppressed the phosphorylation of both Forkhead box O1 (FOXO1) and Forkhead box O 3 A (FOXO3A), which were AKT downstream targets and upstream regulators of apoptotic cascade^20^. These results were further confirmed in Caco-2 and A549 cells (Supplementary Fig. 2b). Furthermore, overexpression of constitutively activated form of AKT (CA-AKT) in Hela cells provided full cell resistance to ConA induced apoptosis, evidenced by levels of CASP9 cleavage, FOXO1 phosphorylation (Fig. 3b), as well as Annexin V-FITC/PI staining (Fig. 3c, Supplementary Fig. 2c). On the other hand, the activities of major critical cell stress sensing molecules, including the phosphorylation levels of 5′ AMP-activated protein kinase (PRKAA1), eukaryotic Translation Initiation Factor 2 A (EIF2A), and I kappa-B kinase-alpha/beta (IKKα/β), as well as the total level of tumor protein p53 (TP53), were not influenced (Fig. 3a).

### Detergent-resistant RTK complex formation by oligomeric ConA

**Fig. 4:**
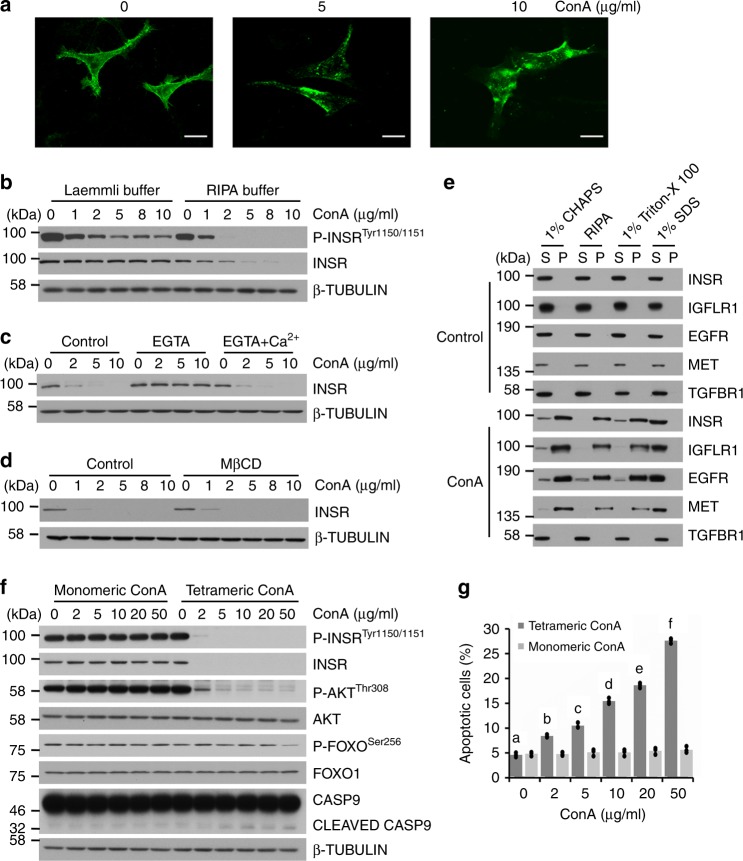
Detergent-resistant RTK complex formation by oligomeric ConA. **a** Hela cells were transfected with hINSR-GFP and treated with ConA for 20 min, and examined under confocal microscopy. Scale bar, 20 µm. **b** Hela cells were treated with ConA (20 μg/ml) for 20 min and stimulated with 100 nM insulin for 15 min, then lyzed with either RIPA buffer, or directly solubilized in hot Laemmli buffer and examined by western analysis. **c** Hela cells were pre-treated with EGTA for 1 h and then treated with 1.8 mM CaCl_2_, the level of INSR was measured by western blot. **d** Hela cells were pre-treated with lipid raft disruptor MβCD for 30 min, the level of INSR was measured by western blot. **e** Hela cells were treated with ConA (20 μg/ml) for 20 min and lyzed with 1% CHAPS, RIPA, 1% Triton X-100 or 1% SDS separately. The level of INSR in supernatant (S) and precipitate (P) was measured by western blot. **f** Hela cells were treated with monomeric ConA or tetrameric ConA for 9 h and stimulated with 100 nM insulin for 15 min. The levels of INSR, AKT, FOXO1, Pro/cleaved CASP9 and the phosphorylation levels of INSR, AKT, FOXO1 were measured by western blot. **g** Annexin V-FITC/PI analysis was done after monomeric ConA or tetrameric ConA treatments. Results were represented as means with standard errors (*n* = 3) and analyzed using one-way ANOVA. Values with different letters (**a**–**f**) in the same column were significantly different (*p* < 0.05) from each other

To understand the mechanism underlying the effects of ConA, we chose INSR as the representative receptor for further characterization. The cellular distribution of INSR was examined by confocal microscopy in Hela cells. As shown in Fig. 4a, the evenly membraneous distributed INSR under control condition was shifted to granulated and clumped patches under ConA treatment, suggesting a possible aggregated status. Furthermore, we found that ConA robustly reduced the solubility of INSR in RIPA buffer in a dose dependent manner (Fig. 4b). Under ConA treatment, EGTA blocked the binding of ConA to INSR and rendered INSR RIPA-soluble, while further calcium supplementation re-imposed the INSR insolubility (Fig. 4c). To examine whether this effect is related to detergent resistant membranes, we treated the cells with the lipid raft disruptor methyl-β-cyclodextrin (MβCD). However, it showed no effect on the receptor solubility changes by ConA (Fig. 4d). As shown in Fig. 4e, not only INSR, but also other receptors, including EGFR, MET and IGFLR1, became insoluble in 1% CHAPS, RIPA, and 1% Triton X-100 after ConA treatment, while these receptors were highly soluble in above detergents in the absence of ConA. Among detergents we screened, only 1% SDS could still solubilize these receptors after ConA treatment. Therefore, we chose 1% SDS as the cell lysis buffer for phospho-tyrosine immunoprecipitation (Fig. 2b). However, the solubility of TGFBR1 in any of the above buffers was not influenced by ConA (Fig. 4e).

It is known that ConA exists as a tetramer at physiological pH and has the potential to form crosslinked complexes^21^. In order to correlate the cellular responses of ConA with its assembly status, ConA was photochemically alkylated so that it became monomerically stable at physiological pH while still retained its intact carbohydrate-binding property, as reported before^22^ and confirmed in the present study (Supplementary Fig. 3a, 3b). As shown in Fig. 4f, in contrast to its tetrameric form, the monomeric ConA showed no effects on the solubilization and phosphorylation of INSR, as well as the activities of intracellular AKT and FOXO1. Monomeric ConA was also not able to induce apoptosis, evidenced by the non-cleavage of CASP9 and Annexin V-FITC/PI staining (Fig. 4g, Supplementary Fig. 3c).

### ConA blocked conformational activation of INSR

**Fig. 5:**
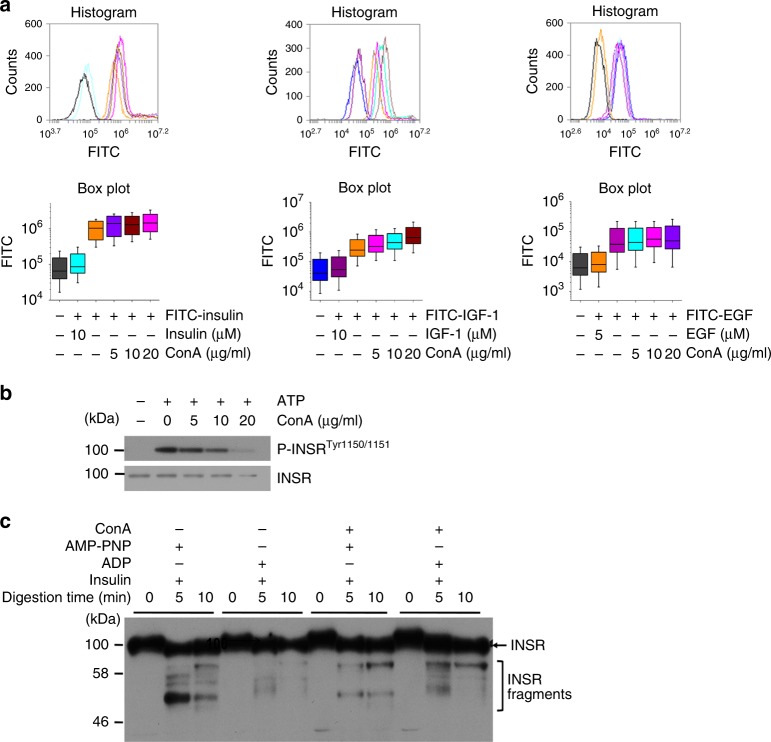
The effects of ConA on ligand binding and conformation of RTKs. **a** The cell surface binding of FITC conjugated insulin, IGF-1, and EGF in the presence of ConA at indicated concentrations. Cells either without ligand addition or coincubated with 10 μM naive were served as controls. The fluorescence-labeled cells were analyzed by flow cytometry. The box plot displays the statistical distribution of FITC fluorescence from the results of the histogram. The horizontal line in the box indicated the median value. The vertical box indicated the 25th or 75th percentile, and the vertical line indicated the maximum or minimum value. **b** Phosphorylated and total INSR in membrane preparations were measured by western blot after being treated with ConA at indicated concentrations and stimulated with 100 nM insulin for 15 min. **c** Membrane preparations were incubated with 20 μg/ml ConA for 20 min and then incubated with 100 nM insulin, 1 mM ADP or AMP-PNP for 15 min. Trypsin was added at 200 μg/ml. Samples were collected at different times points and the cleavages of INSR were examined by western blot using an antibody against the loop region of INSR

The cell surface binding of FITC conjugated insulin, IGF-1 and EGF to their receptors was quantitated by flow cytometry. As shown in Fig. 5a, these ligand bindings were not influenced by ConA. We also showed that not only in cells, but also in membrane preparation ConA was able to inhibit INSR phosphorylation (Fig. 5b). Using the same membrane preparation, we further probed the conformational dynamics of INSR by limited trypsin digestion. As shown in Fig. 5c, INSR was inaccessible to trypsin cleavage in the presence of insulin and ADP. However, the presence of insulin and AMP-PNP enabled the transition of INSR from inactive to active form, which was reported to be liable for trypsin digestion^23,24^ and confirmed in the present study. We further demonstrated that the presence of ConA inhibited this conformational activation.

## Discussion

ConA has been reported to induce apoptosis with DNA fragmentation, mitochondrial depolarization and increased ROS production in multiple cancer cell lines^25,26^. In the present study, we further confirmed its apoptotic effect in Hela, Caco-2, and A549 cell lines. BCL2 family proteins regulate apoptosis by controlling the permeabilization of the outer mitochondrial membrane^27^. The anti-apoptotic BCL2 protein inhibits cytochrome c release through the mitochondrial pore and downstream activation of caspase cascade, while dephosphorylated BCL2-associated death promoter (BAD) binds and inactivate BCL2, thus initiating apoptosis^28^. Our results showed that ConA reduced both BCL2 and phospho-BAD levels. Similar results were reported in human breast carcinoma MCF-7 cells and other cell types^18^. Caspase-mediated apoptosis follows two main pathways, intrinsic and extrinsic^28^. For intrinsic apoptosis pathway, formation of apoptosome and activation of CASP9 will cleave and activate executioner caspases such as CASP3^29^. This leads to degradation of cellular components for apoptosis and results in the characteristic morphology of apoptosis^28^. Consistent with previous report in human melanoma A375 cells^30^, we demonstrated that ConA stimulated cleavage and activation of both CASP9 and 3. On the other hand, the activation/self-cleavage of CASP8 is a hallmark for the execution of extrinsic apoptotic signaling pathway^28,29^. In the present study, we did not observe the activation of CASP8 by ConA treatment. Therefore, ConA selectively stimulate intrinsic but not extrinsic apoptosis pathway. ConA was also reported to be able to induce autophagy but with prolonged treatment (>12 h)^31^. Indicated by autophagic markers including BECN1 and ATG12, we did not observe obvious autophagy in the experimental conditions we used (9 h of ConA treatment).

ConA’s biological activities are closely associated with its binding to cell surface receptors with high α-D-mannosyl and α-D-glucosyl moieties^32^. It preferentially agglutinates cells transformed by oncogenic viruses more than their untransformed counterparts and inhibits growth of malignant cells in animals^33^. These properties make it an attractive object for further investigation for potential anti-cancer therapies. To date, most of the studies were conducted on the characterization of ConA’s effect on apoptotic biomarkers^28^. However, much less were reported on the influences of upstream signaling pathways that led to these apoptotic effects. As shown in the present study, ConA was found to selectively bind to RTKs including INSR, IGFLR1, EGFR, and MET, but not with TGFBR1, TNFR1 and DR4. Moreover, the present study clearly demonstrated that ConA reduced the phosphorylation levels of its associated RTKs, but not other cell surface receptors. EGTA is known to abolish the binding of ConA to carbohydrates^34^. The presence of EGTA abolished the apoptotic effects of ConA (Fig. 2d, e). These results demonstrated that the apoptotic effect of ConA was dependent on its interactions with cell surface receptors. The selective inhibition of these receptors should underlie its specific induction of intrinsic but not extrinsic apoptosis, which is associated with activation of receptors including TNFR1 and DR4^28^.

Appropriate RTK signaling is critical for cell survival and proliferation, while aberrant activation of RTKs and their intracellular signaling pathways have been causally linked to cancers, diabetes, inflammation, et al.^2^. The downstream of cell surface RTKs is a plethora of intracellular signaling molecules that form interconnected networks to receive signals from RTKs and process into combinatorial outcomes^35^. Understandably, due to cross-talk mechanisms, inhibition of one RTK leads to compensation of the other RTK or downstream signaling have been observed and suggested to be a major cause of drug resistance^36^. Therefore, a combinatorial inhibition of multiple RTKs and/or downstream components may have a better effect in cancer treatment and is much needed^37^. Among the downstream of cell surface RTK signaling networks, mTOR, MAPK and PI3K/AKT signaling cascades are the most critical for cell survival and the favorable drug targets^38^. mTOR is a master regulator of cellular metabolism through multiple pathways activated by intracellular nutrients and extracellular growth factors and hormones^39^. MAPK1/2 cascade responds to the cell surface activation of RTKs and promotes cell survival by either downregulating the activity or levels of pro-apoptotic molecules, or upregulating anti-apoptotic molecules^40^. In the present study, the activities of both mTOR and MAPK1/2 signaling were down regulated by ConA. However, the most prominent signaling change after ConA treatment was the inhibition of AKT phosphorylation. The PI3K-AKT pathway receives signals from multiple RTKs^41^ and is essential for cell survival as activated AKT influences many factors involved in apoptosis, either by transcription regulation or direct phosphorylation^42^. Once activated, AKT exerts anti-apoptotic effects through phosphorylation of substrates that directly regulate the apoptotic machinery such as BAD or CASP9, or phosphorylation of substrates that indirectly inhibit apoptosis such as forkhead transcription family members^43^. Accordingly, ConA was also found to reduce the phosphorylation of BAD and FOXO1 (Figs. 1b, 3a). Our results further demonstrated that cells overexpressing constitutively activated AKT became resistant to ConA-induced apoptosis. This result clearly showed that AKT was the key molecule that bridged upstream ConA- mediated RTK inhibition and downstream apoptosis initiation. A plethora of studies have demonstrated that AKT is frequently overexpressed and activated in a variety of human cancers, including lung, breast, ovarian, gastric and pancreatic carcinomas^44–46^. Inhibition of AKT activities thus represents an important approach for drug development^47^.

A pitfall for multiple targeted therapies is almost always associated with indiscriminative signaling inhibition and cytotoxicity^5,7,48^, which generally cause imbalance of the cellular homeostasis commonly designated as cellular stress. This in turn is sensed by a repertoire of intricate signaling checkpoints that are wired to mitochondrial dynamics and cell death machinery^49^. In particular, indicated by the EIF2A (eukaryotic initiation factor 2A) phosphorylation levels, ER stress is known to respond to environmental stresses to manage cellular injury or alternatively induce apoptosis by inducing changes in the abundance of BCL2 proteins^50^. PRKAA1 acts as a primary sensor of mitochondrial stress and ATP production^51^. Being a central regulator of cellular stress responses, NFKB is governed by the phosphorylation of IKKα/β complex and the stability of I-κB proteins, which determines the efficiency of NFKB mediated expression of inflammation and survival genes^52^. Furthermore, transcription factors such as TP53 have been shown to control the transition between the rapid and delayed phase of stress responses that possibly lead to autophagy and/or apoptosis^53^. In the present study, these stress-related checkpoints were examined and no immediate perturbations were found by ConA treatment. These results thus highlighted the specificity of ConA on intracellular signaling. It should be noted that the present study was designed to treat the cells for limited time (4 h) to elucidate the immediate signaling influenced by ConA. There was study showed that ConA reduced NFKB and increased TP53 levels in MCF-7 cells after high concentrations (up to 100 µg/ml) and prolonged (24 h) treatment^26^. Based on our results, these effects should be indirect.

An unexpected finding in the present study was that the RTKs that were bound and inhibited by ConA became resistant to multiple cell lysis buffers including 1% CHAPS, 1% Triton X-100, and RIPA after ConA treatment (Fig. 4e). This effect was selective because receptors such as TGFBR1 were not influenced. Given the fact that ConA adopted tetrameric form at physiological pH and was able to oligomerize glycoproteins such as quail ovalbumin in biochemical preparations^19^, it might be possible that ConA crosslinked cell surface RTKs and rendered them detergent resistant. The monomeric form of ConA was prepared and no longer influenced the solubility and activities of RTKs (Fig. 4f). The monomeric form of ConA also exerted no apoptotic effects to cells (Fig. 4g). These results demonstrated that the above cellular effects of ConA were dependent on its oligomeric status.

To further understanding the mechanism of ConA mediated inhibition of RTKs, we examined the possible influences of ConA on ligand binding of several RTKs. Previous studies have demonstrated that ConA did not influence the binding of insulin to its receptor^54^, as further confirmed in the present study (Fig. 5a). We also demonstrated that neither IGF1 nor EGF binding to their receptors were influenced by ConA. Therefore, the inhibition of RTKs by ConA was not likely due to impaired ligand binding. On the other hand, the molecular mechanisms underlying activation of RTKs share common themes. In general, ligand binding induces conformational changes involving activation of the intracellular tyrosine kinase domain and autophosphorylation^55^. As a typical example, the active and inactive conformation of INSR are mainly due to the orientations of the activation loop, which blocks substrate access to the catalytic cleft in the basal/inactive state while swings away from the orientation of the inactive conformation to form a platform for substrate binding^56^. As demonstrated by previous studies^23^ and confirmed in the present study (Fig. 5c), this conformational change could be readily detected by limited trypsin digestion: the activation loop of INSR is susceptible for trypsin cleavage upon activation through ligand and ATP binding but only very weakly in its basal state. We further demonstrated that ConA readily inhibited the trypsin cleavages of INSR in the presence of insulin and AMP-PNP. This might suggest that ConA blocked this conformational transition from inactive to active state. Further understanding the conformational dynamics of INSR and other RTKs under ConA crosslinking should be critical for the manipulation of activities of these receptors.

A key feature of cancer cell metabolism is high rate of glucose uptake to cope with the increased energetic and biosynthetic needs^57^. The abundance of glucose in the cytoplasm of cancer cells not only contributes to increased glycolysis but also increases flux into the metabolic branch pathways, such as the hexosamine biosynthetic pathway (HBP), which provides substrates for N- and O-linked glycosylation^58^. In a cancer context, the high levels of β1, 6-branched N-glycans destabilizes cell adhesion and favors the activation of IGFLR1 mediated signaling, thus increasing the downstream AKT signaling and promote tumor progression^59^. Inhibition of N-linked glycosylation has been shown to markedly reduce RTK signaling and radiosensitize tumor cells^60^. Notably, the extracellular domain of receptors for insulin, IGF-1, EGF, etc. that stimulate cell proliferation and oncogenesis are highly glycosylated^61^, therefore associated with ConA lattice and became detergent resistant and inhibited. Conversely, growth-arrest receptors involved in organogenesis and differentiation (such as TGFBR1) have few *N*-glycan sites and are not associated with ConA lattice.

ConA is known not only to preferentially bind and induce apoptosis in cancerous cells, but also to simultaneously stimulate cytokine production and activate the immune system for eradication of the tumor^18^. Such dual character of immunomodulating and apoptosis-inducing activities has drawn much attention for anti-tumor studies^18^. The mechanism discovered in the present study should provide valuable information for further studies toward this direction. Although there have been evidences showing that ConA could discriminate between malignant and non-malignant cells^62^, it should be acknowledged that ConA was known to cause liver injury and less likely to directly serve as a drug^63^. Further development of tailor-made compounds that are highly selective to crosslink and inhibit RTKs of tumor cells may represent another strategy for anti-cancer therapies.

## Methods

### Cell culture and treatment

The human cervical cancer Hela cells, colon adenocarcinoma Caco-2 cells, lung cancer A549 cells were obtained from the Type Culture Collection of the Chinese Academy of Sciences, Shanghai, China. Hela cells were maintained in DMEM (Invitrogen, # 10569010) with 10% fetal bovine serum (Invitrogen, # 10099141) and 1% antibiotics (penicillin 100 U/ml, streptomycin 100 μg/ml) at 37 °C under 5% CO_2_ in a humidified incubator. Caco-2 cells were maintained in Eagle’s Minimum Essential Medium (Invitrogen, # 11095080), 20% fetal bovine serum, 1 mM sodium pyruvate (Invitrogen, #11360088), 2 mM glutamine (Invitrogen, # 25030081) and 1% antibiotics in a humidified atmosphere of 5% CO_2_ in a humidified incubator. A549 cells were maintained in Ham’s F-12K (Invitrogen, # 21127022) with 10% fetal bovine serum and 1% antibiotics at 37 °C under 5% CO_2_ in a humidified incubator. The cells were tested for mycoplasma contamination.

For cell transfections, the plasmids used in the present study were obtained from Addgene, including wild-type AKT (Addgene plasmid # 9021), constitutively actived AKT (T308D S473D, Addgene plasmid # 14751), and hINSR-GFP (Addgene plasmid # 22286). Cells were transfected using lipofectamine 3000 (Thermo, #L3000001) according to the manufacturer’s protocol. Cells were subjected to further analyses after 48 h of transfection. For compound treatments, cells were incubated with lipid raft disruptor MβCD (Sigma, # 332615) at 10 mM in DMEM for 30 min before further treatment with ConA at indicated concentrations. In separate experiments, cells were co-incubated with ConA and 5 mM EGTA in DMEM for 20 min.

### Cell apoptosis assay

Cell apoptosis was measured using an Annexin V-FITC/propidium iodide (PI) apoptosis detection kit (Beyotime, # C1062) following the manufacturer’s instructions. Briefly, Hela cells were treated with a series of concentrations of ConA (Sigma, #C5275) at 0, 2, 5, 10, 20 or 50 μg/ml in 6-well plates for 9 h. Cells were then trypsinized and centrifuged at 1000 rpm for 5 min. The cell pellet was washed with PBS once and re-suspended with 195 μl binding buffer plus 5 μl Annexin V-FITC for 10 min, and stained with 10 μl PI for 10 min. Then, the samples were diluted with 400 μl binding buffer and approximately 10, 000 cells from each sample were analyzed with a BD Accuri™ C6 flow cytometer. The percentage of cells positive for Annexin V-FITC and/or PI was reported inside the quadrants.

### Biotinylated ConA pull-down assay

Cells were lyzed in 50 mM HEPES, 10 mM MgCl_2_, 0.1% Triton X-100, 150 mM NaCl with protease inhibitor cocktail. After pre-cleared with MyOne™ Streptavidin T1 beads (Thermo, # 65601) for 1 hr, cell lysates were then co-incubated with biotinylated-ConA (0.15 mM) (Vector, # B1005) and MyOne™ Streptavidin T1 beads for 2 h at 4 °C with rotation. The beads were then washed three times with ice-cold lysis buffer and captured proteins were eluted by Laemmli buffer for western blot analysis. The beads pre-blocked with biotin (0.15 mM) (Sigma, # B4501) served as the control.

### Cell signaling analyses

To examine the effects of ConA on intracellular and cell surface signaling, cells at ~90% confluency were treated with designated concentrations of ConA (in DMEM, MEM, Ham’s F-12K for Hela, Caco-2, and A549 cells respectively) for 4 h after the full medium was removed. Subsequently, cells were replenished with full medium plus ConA for 15 min for cell signaling activations. The activities of intracellular signaling molecules were examined by western blot using the correlated antibodies. For analysis of cell surface receptor tyrosine kinase activities, cells were lyzed with 50 mM Tris, 150 mM NaCl, 1% SDS with protease and phosphatase inhibitors for 20 min and diluted with equal volume of RIPA lysis buffer (50 mM Tris, 150 mM NaCl, 0.5% NP-40, 0.1% SDS, 1 mM EDTA, pH 7.4, with protease and phosphatase inhibitor cocktail (Roche)). For phosphotyrosine immunoprecipitation, Hela cell lysates were pre-cleared with MyOne™ Streptavidin T1 beads (Thermo, # 65601) at 4 °C for 2 h and then immunoprecipitated with a biotinylated phospho-tyrosine antibody (PY20) (Gibco, # MA12439) at 4 °C overnight. The immunoprecipitated proteins was captured with 30 μl magnetic beads for 1 h at 4 °C and washed three times with ice-cold RIPA buffer. The captured proteins were then eluted from beads with Laemmli buffer and heated at 95 °C for 5 min for western blot analysis.

### Confocal microscopy analysis of INSR

For confocal microscopy analysis, Hela cells were seeded onto poly-lysine coated coverslips (Corning, # 354086) and transfected with hINSR-GFP (Addgene plasmid # 22286) using lipofectamine 3000 following the manufacturer’s protocol. After 48 h of transfection, cells were treated with ConA at 0, 5, 10 μg/ml for 20 min and fixed with 4% paraformaldehyde (Sigma, # P6148) for 20 min at room temperature. The coverslips were then washed three times with PBS, mounted on microscope slides and visualized by confocal microscopy using a Nikon A1 laser scanning confocal microscope. All experiments were repeated at least three times.

### Detergent solubility analysis of cell surface receptors

For detergent solubility analysis, Hela cells were treated with or without ConA at 20 μg/ml for 20 min and rinsed twice with ice-cold PBS. Afterwards, cells were lyzed in 50 mM Tris, 150 mM NaCl, with 1% CHAPS, 1% Triton X-100, or 1% SDS separately with protease inhibitor cocktail for 1 h at 4 ^o^C. Otherwise, cells were lyzed with RIPA buffer (50 mM Tris, 150 mM NaCl, 0.5% NP-40, 0.1% SDS, 1 mM EDTA, pH 7.4, with protease inhibitor cocktail (Roche)). Cell lysates were cleared by centrifugation at 12,000 g for 20 min at 4 °C. The supernatants were collected and added with equal volume of 2x Laemmli buffer. The pellets were solubilized with the same total volume of hot Laemmli buffer as the supernatant did. Both the supernatants and the solubilized pellets were subjected for western blot analysis of cell surface receptors.

### Preparation of monomeric ConA

Monomeric ConA was prepared and purified according to the procedure described before with minor modifications^22^. Specifically, a solution was prepared with 10 mM Tris, 1 M NaCl, 1 mM MnCl_2_, 1 mM CaCl_2_, 10 mM methyl α-D-mannopyranoside, 100 mM chloroacetamide, pH 7.3. This solution was deoxygenated under argon for 60 min and used to dissolve ConA to a final concentration of 1.5 mg/ml. The ConA solution was irradiated with a high-pressure mercury lamp (1000 W) for 90 min and then dialyzed against 0.1 M Tris to remove methyl mannose. The solution was concentrated by ultrafiltration (Amicon, MW cutoff 10, 000 Da) and passed through a column of Superdex 200 Increase 10/300 GL using 50 mM Tris, 150 mM NaCl, 100 mM glucose as the mobile phase. The elution of different forms of ConA was monitored with UV absorption at 280 nm and determined by SDS-PAGE. The molecular weight was calibrated using a gel filtration calibration kit (GE Healthcare, # 28403841). The purified monomeric and tetrameric ConA was further dialyzed against 10 mM sodium acetate, 1 mM MnCl_2_ and 1 mM CaCl_2_ pH 4.6, and concentrated by ultrafiltration before they were used for further experiments.

### Ligand binding assay

The cell surface ligand binding assay was conducted as described before^64^. Specifically, Hela cells were grown in 10 cm dishes to approximately 70% confluency and treated with ConA at 0, 5, 10, 20 μg/ml for 20 min. Subsequently, 1 μM FITC-labeled insulin or IGF-1 (produced by Shanghai Top-peptide Biotechnology) was added with or without 10 μM native insulin or IGF-1 for 30 min to reach equilibrium of ligands binding^65^. For EGF binding, A549 cells were treated with ConA at indicated concentrations and incubated with 0.5 μM FITC-EGF in the presence or absence of 5 μM naive EGF for 30 min. Cells were washed three times with ice-cold PBS and analyzed using a BD Accuri™ C6 flow cytometer. All experiments were repeated at least three times.

### Cell membrane preparation

The crude membrane preparation was conducted as described previously^66^. Briefly, after washed twice with ice-cold PBS, Hela cells were scrapped and pelleted through centrifugation at 5000 rpm for 5 min. Cells were then resuspended and homogenized in 10 mM HEPES, 250 mM sucrose, 5 mM MgCl_2_, 5 mM CaCl_2_ with protease and phosphatase inhibitors using a glass Dounce homogenizer, followed by centrifugation at 5000 rpm for 5 min. The supernatant was collected and further centrifuged at 100,000 g for 2 h using a HITACHI CS150GX II ultracentrifuge. The membrane pellets were collected and stored at −80 °C for further experiments.

### In vitro autophosphorylation of INSR

The in vitro activation and autophosphorylation of INSR was conducted following the previously published protocol^67^. Briefly, membrane pellets prepared above were homogenized in reaction buffer (10 mM Tris, 5 mM MgCl_2_, 5 mM MnCl_2_, 0.1% Triton X-100, pH 7.4) with protease and phosphatase inhibitor cocktail. The homogenate was then treated with ConA at 0, 5, 10, 20 μg/ml and stimulated with 100 nM insulin and 1 mM ATP (Sangon, # A600020-0005) at room temperature for 15 min. The samples were then added with RIPA buffer for western blot analysis. Reactions without ATP supplementation were used as controls.

### Limited trypsin digestion

The limited trypsin digestion was conducted as described before with minor modifications^23^. The membrane pellets prepared above were homogenized with reaction buffer (10 mM Tris, 10 mM MgCl_2_, 5 mM MnCl_2_, 0.1% TritonX-100, pH 7.4) and incubated with 20 μg/ml ConA for 20 min. Otherwise, samples without ConA were incubated with 20 μg/ml of BSA were served as controls. Afterwards, the samples were then incubated with 100 nM insulin, 1 mM ADP (Sangon biotech, # A610016) or AMP-PNP (Sigma, # A2647), and then trypsin (Sigma, # T6567) was added at a final concentration of 200 μg/ml. The reactions were conducted at 25 °C and stopped at different time points by Laemmli buffer. The cleavages of INSR were examined by western blot using an antibody against the activation loop of INSR (CST, # 3025).

### Western blot analysis

For western blot analysis, cells were rinsed twice with ice-cold PBS and lyzed with the indicated lysis buffers. The cell lysates were collected after centrifugation at 12,000 g for 20 min at 4 °C. Protein concentration was determined by a BCA Protein Assay Kit according to the manufacturer’s instructions. After normalization, samples (10 µg of protein) were separated by SDS-PAGE gel for 1 h at 150 V and then transferred to a 0.45-µm polyvinylidene difluoride membrane (PVDF) (Millipore) for 1 h at 100 V. After transfer, the membrane was blocked with 5% nonfat milk in TBST for 1 h and incubated with primary antibodies (1:1000) overnight at 4 °C, followed by secondary antibodies for 1 h, and developed with ECL Reagents (Beyotime Biotechnology). The following antibodies were used: antibodies against BCL2 (CST, # 4223), phospho-BAD (Ser136) (CST, # 4366), BAD (CST, # 9239), CASP9 (CST, # 9502), CASP3 (CST, # 9665), CASP8 (CST, # 9746), BECN1 (CST, # 3738), ATG12 (CST, # 4180), phospho-INSR (Tyr1150/1151) (CST, # 3024), INSR (CST, # 3020), IGFLR1 (CST, # 3027), EGFR (CST, # 2232), MET (Santa Cruz, # 8057), phospho-TGFBR1 (Thermo, # PA5-40298), TGFBR1 (Santa Cruz, # 518018), TNFR1 (Santa Cruz, # 8436), DR4 (Santa Cruz, # 8411), phospho-Tyr (PY20) (Thermo, # MA12439), phospho-AKT (Thr308) (CST, # 13038), phospho-AKT (Ser473) (CST, # 3787), AKT (CST, # 9272), phospho-MAPK1/2 (Thr202/Tyr204) (CST, # 4370), MAPK1/2 (CST, # 9107), phospho-RPS6KB1 (Thr389) (CST, # 9205), RPS6KB1 (CST, # 9202), phospho-FOXO1 (Ser256) (CST, # 9461), FOXO1 (CST, # 2880), phospho-FOXO3A (Ser253) (CST, # 9466), FOXO3A (CST, # 2497), phospho-PRKAA1 (Thr172) (CST, # 50081), PRKAA1 (CST, # 5831), phospho-EIF2A (Ser51) (CST, # 3597), EIF2A (CST, # 9722), TP53 (CST, # 2527), phospho-IKKα/β (Ser176/180) (CST, # 2679), CHUK (CST, # 11930), IKBKB (CST, # 8943), HA-tag (CST, # 3724), β-TUBULIN (CST, # 2146). The densities of the protein bands were normalized to that of β-TUBULIN, which served as an inner control. All the band intensities were quantified using NIH Image 1.63 software. Full size western blot images are shown in Supplementary Figs. 4 to 8.

### Statistics and reproducibility

All statistical evaluations were analyzed by one-way analysis of variance (ANOVA) followed by Tukey’s HSD post hoc test using the software SPSS 19.0. Tukey’s HSD post hoc test was used to examine treatment differences among the interactions. When the interaction was significant, the results were further analyzed using one-way ANOVA and Turkey’s HSD post hoc test. In case unequal variance was determined by Levene’s test, the data were square root-transformed before statistical analysis. A value of *P* < 0.05 was considered statistically significant. Each value is expressed as means ± S.E.M. All experiments were repeated at least three times.

### Reporting summary

Further information on research design is available in the Nature Research Reporting Summary linked to this article.

## Supplementary information

Supplementary information

Description of Additional Supplementary Files

Supplementary Data 1

Reporting Summary

## Data Availability

The data that support the findings of this study are available from the corresponding author on reasonable request. The source data underlying plots are provided in Supplementary Data 1.
