# Supplementary material for: Apoptosis of cancer cells is triggered by selective crosslinking and inhibition of receptor tyrosine kinases
Source: Commun Biol. 2019 Jun 21;2:231. doi: 10.1038/s42003-019-0484-5 (PMC6588694; doi:10.1038/s42003-019-0484-5)
Supplement: Supplementary file 1 — Supplementary information [file 42003_2019_484_MOESM1_ESM.pdf]

Supplementary Figure 1

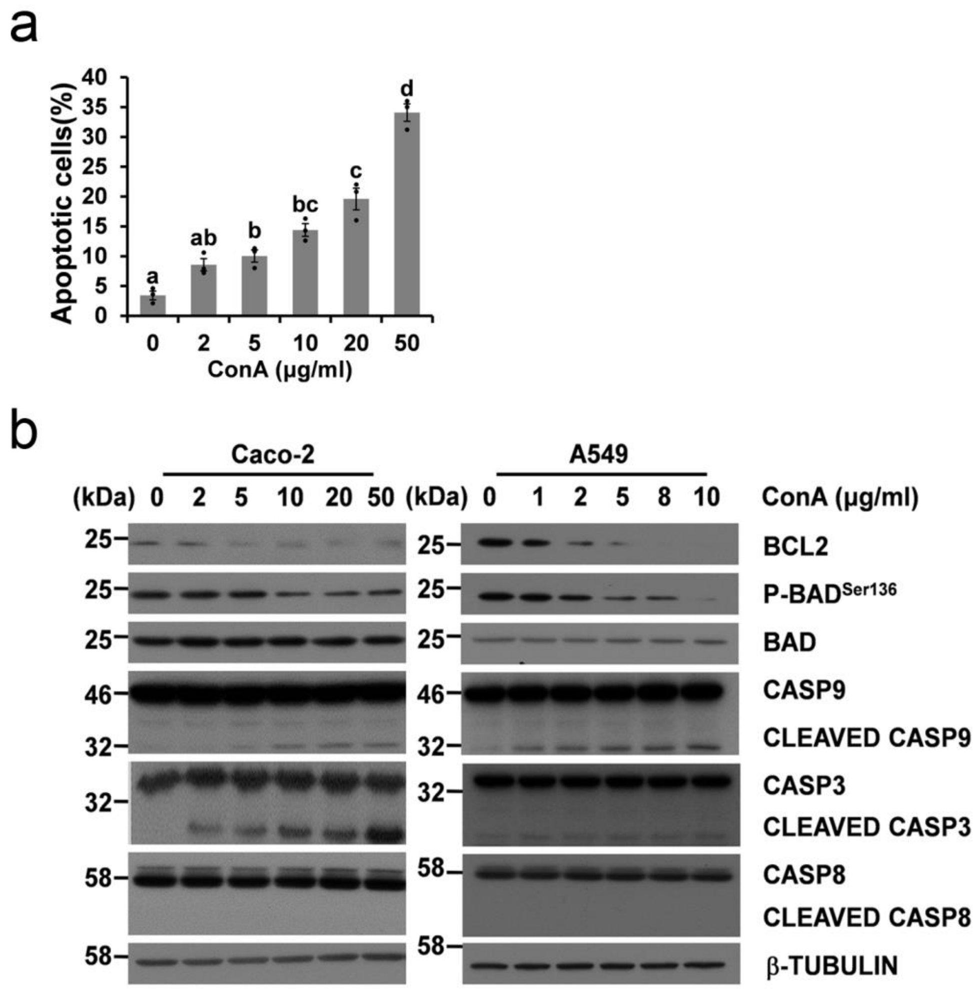

Supplementary Figure 1. ConA induced apoptosis in cancerous cell lines. (a) The quantitative data of Figure 1a. **(b)** Caco-2 and A549 cells were treated with ConA for 9 hrs at indicated concentrations. The levels and activities of proteins involved in apoptosis were examined by western blot. Results were represented as means with standard errors (n=3) and analyzed using One-Way ANOVA. Values with different letters (a-d) in the same column were significantly different ($p < 0.05$) from each other.

Supplementary Figure 2

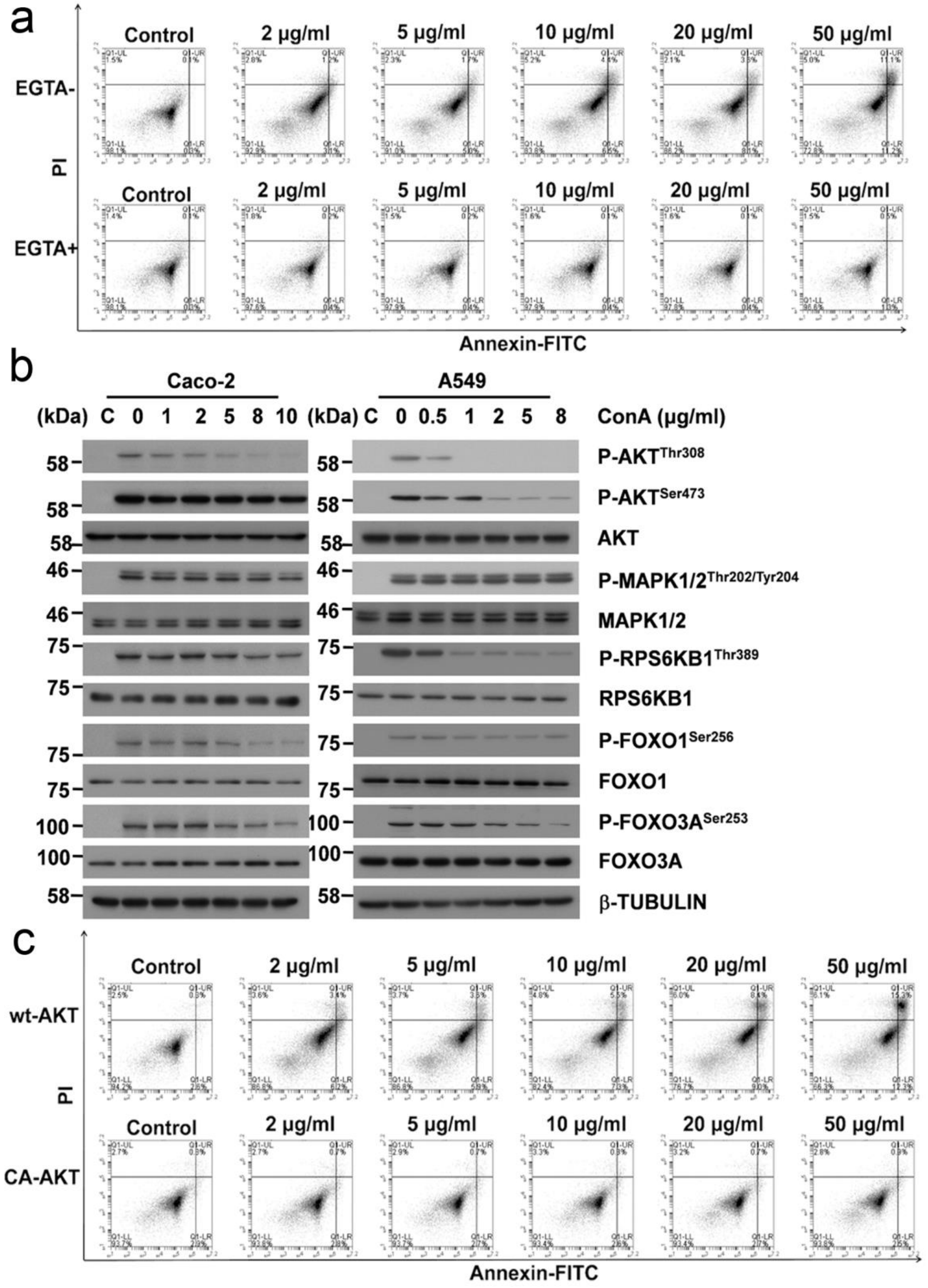

Supplementary Figure 2. The influences of ConA on intracellular signaling. (a) Annexin V-FITC/PI analysis was done in Hela cells after co-incubation of 5 mM EGTA and ConA at indicated concentrations for 9 hrs. **(b)** Caco-2 and A549 cells were treated with ConA

for 4 hrs and stimulated with full medium for 15 min. The levels and activities of key signaling molecules involved in survival pathways were examined by western blot. **(c)** Hela cells were transfected with wide-type or constitutively activated AKT, then treated with ConA for 9 hrs and stained with Annexin V-FITC/PI for apoptosis analysis using flow cytometry.

Supplementary Figure 3

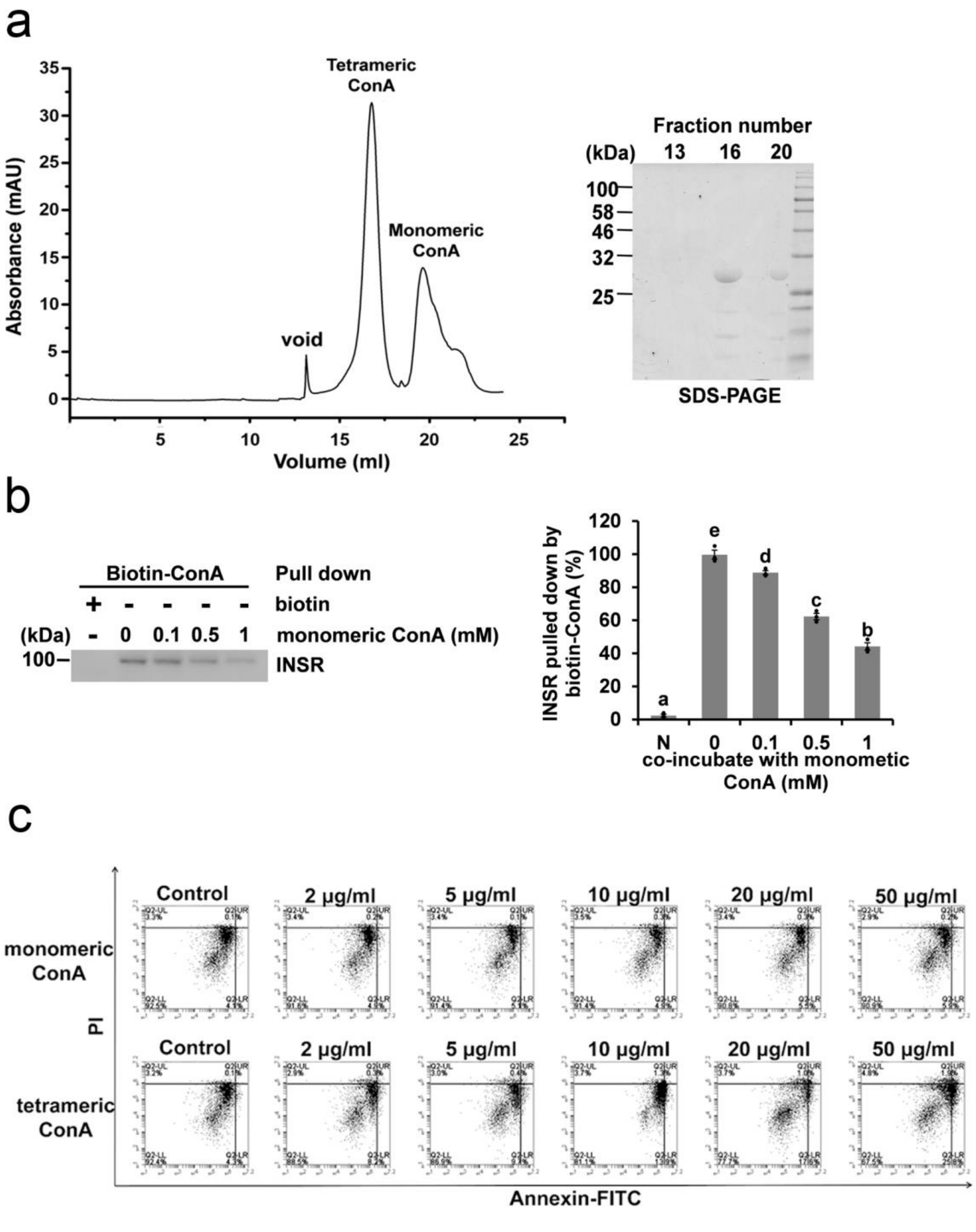

Supplementary Figure 3. The preparation and property of monomeric ConA. (a) Elution profile of different forms of ConA through a Superdex 200 Increase 10/300 GL column followed by SDS-PAGE. Fraction numbers were shown at the top. **(b)** The INSR binding property of monomeric ConA was demonstrated by a competition assay. Membrane preparations were treated with monomeric ConA at 0, 0.1, 0.5, 1 mM for 1 hr at 4 °C, and

further incubated with 0.1 mM biotinylated-Co A for 1 hr at 4 °C. IR was pulled down using MyOne™ Streptavidin T1 beads and examined by western blot and quantitated. Monomeric ConA successfully competed the binding of INSR to biotin-ConA. **(c)** Annexin V-FITC/PI analysis was done after monomeric ConA or tetrameric ConA treatment. Results were represented as means with standard errors (n=3) and analyzed using One-Way ANOVA. Values with different letters (a-e) in the same column were significantly different ($p < 0.05$) from each other.

Supplementary Figure 4

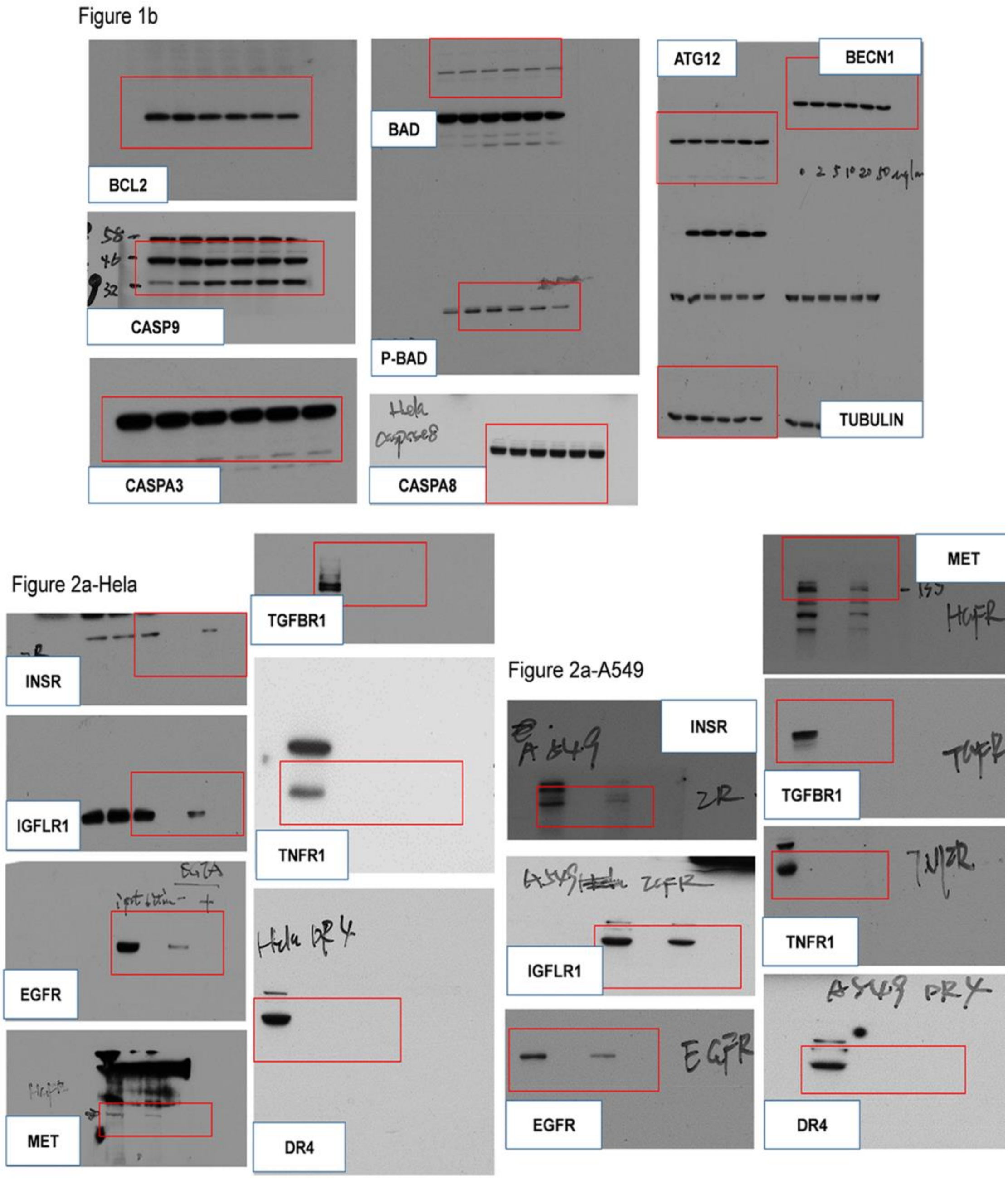

Supplementary Figure 4. Full western blot images of Figure 1b and 2a.

Supplementary Figure 5

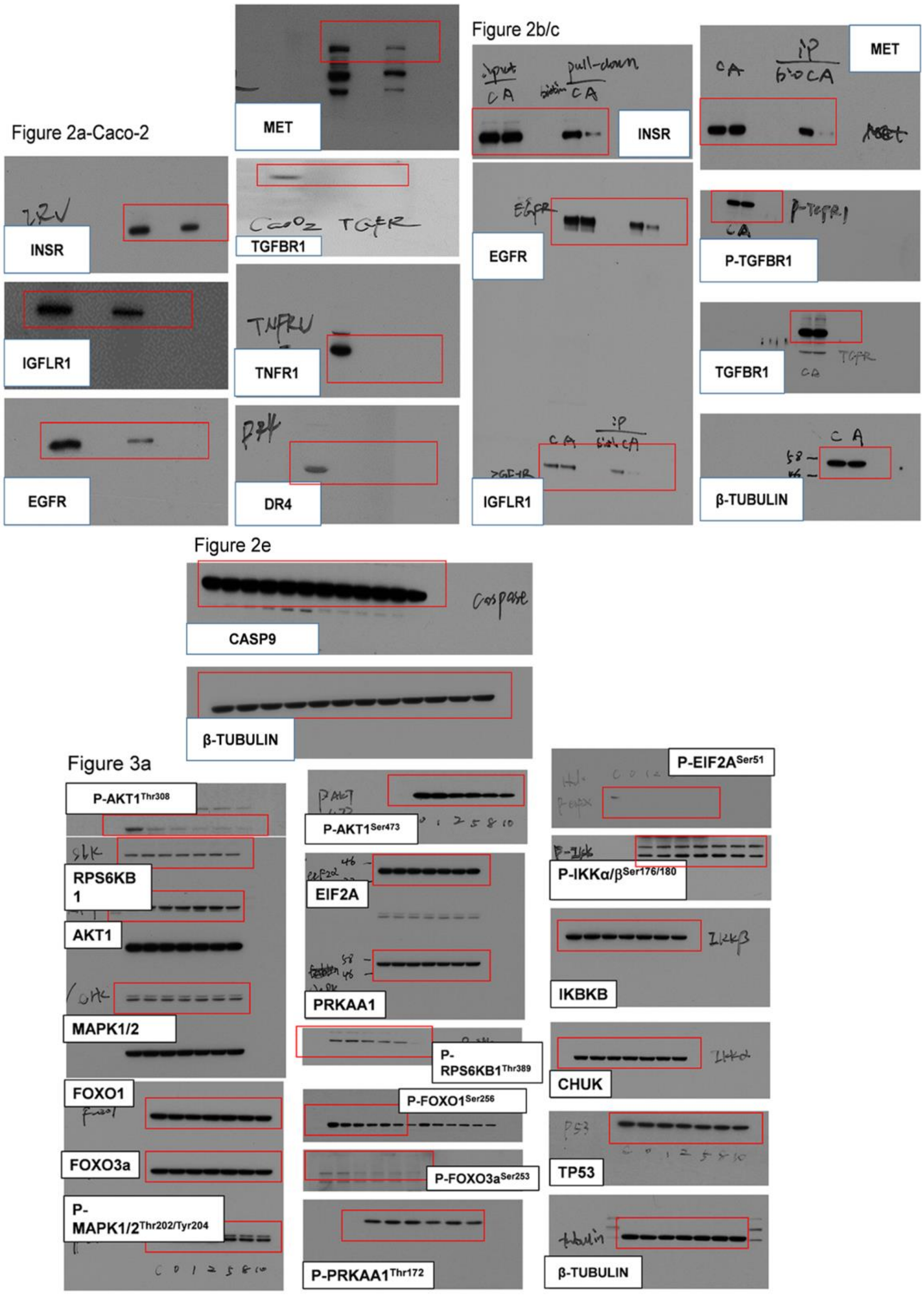

Supplementary Figure 5. Full western blot images of Figure 2a, 2b, 2c, 2e and 3a.

Supplementary Figure 6

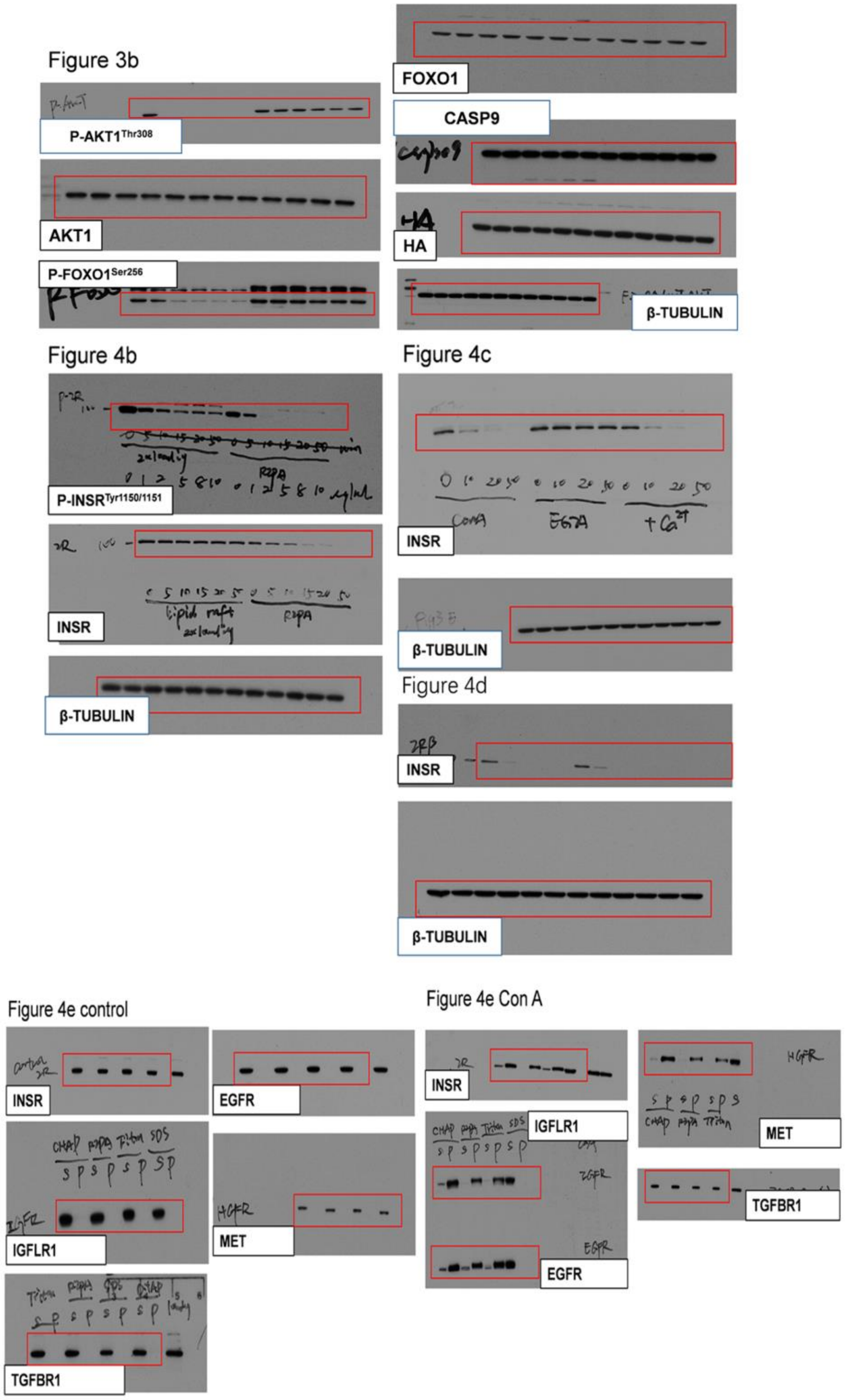

Supplementary Figure 6. Full western blot images of Figure 3b, 4b, 4c, 4d and 4e.

Supplementary Figure 7

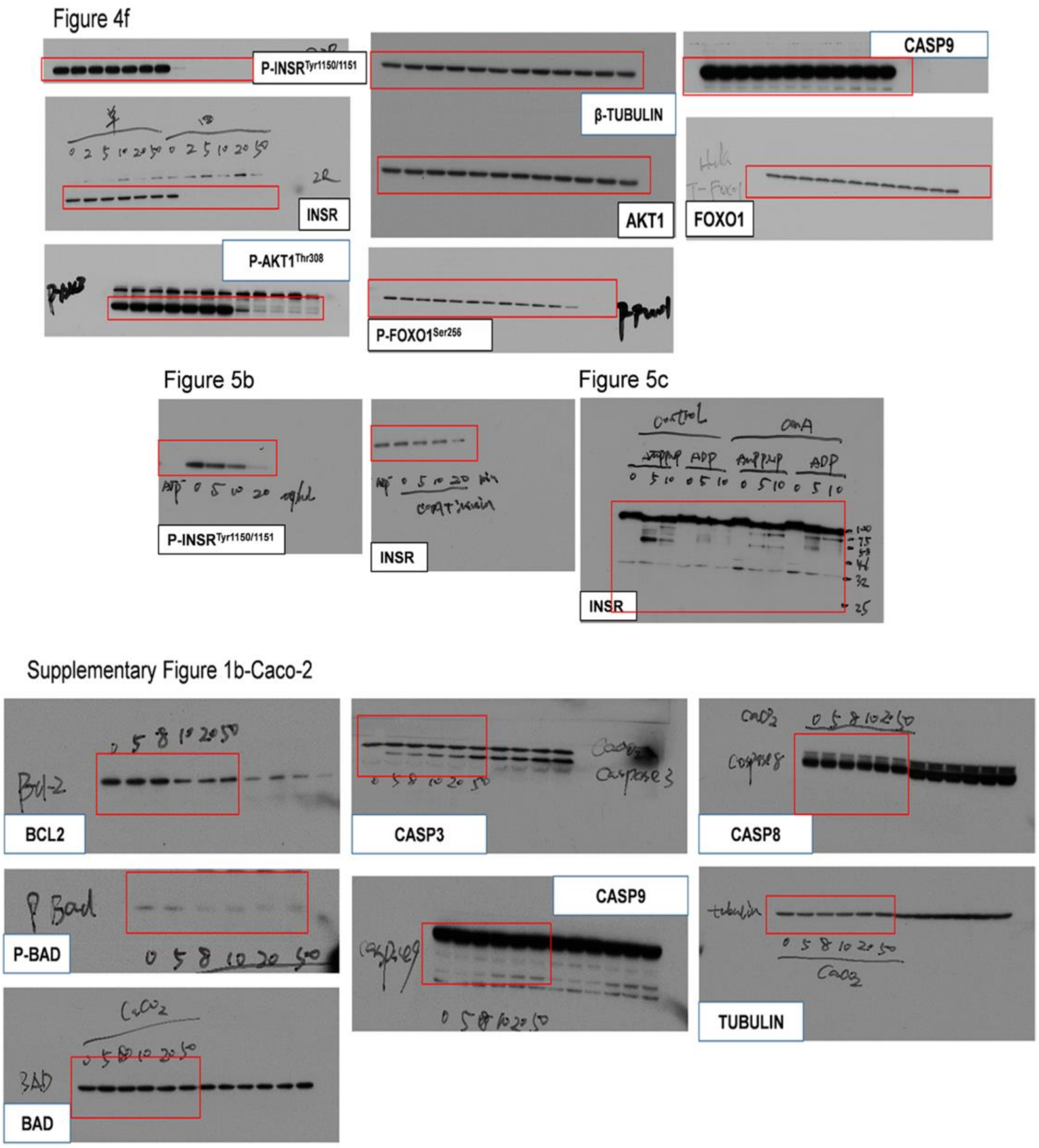

Supplementary Figure 7. Full western blot images of Figure 4f, 5b, 5c, and Supplementary Figure 1b.

Supplementary Figure 8

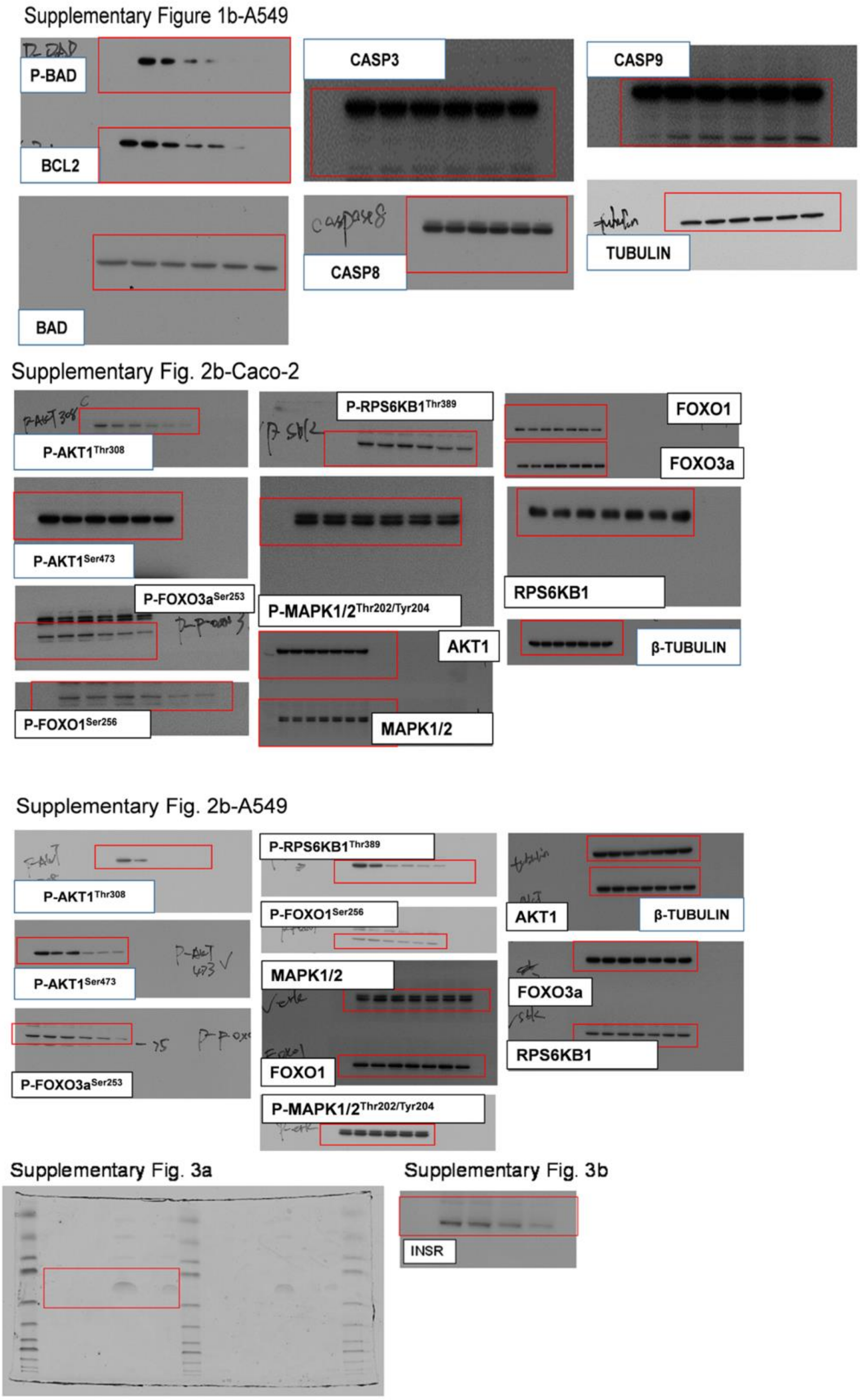

Supplementary Figure 8. Full western blot images of Supplementary Figure 1b, 2b 3a and 3b.
