# Supplementary material for: Apoptosis of cancer cells is triggered by selective crosslinking and inhibition of receptor tyrosine kinases
Source: Commun Biol. 2019 Jun 21;2:231. doi: 10.1038/s42003-019-0484-5 (PMC6588694; doi:10.1038/s42003-019-0484-5)
Supplement: Supplementary file 2 — Description of Additional Supplementary Files [file 42003_2019_484_MOESM2_ESM.docx]

**Description of Additional Supplementary Files**

**File Name**: Supplementary Data 1

**Description**: Source data underlying plots (Figure 1 - Supplementary Figure 3).
