# Supplementary material for: Apoptosis of cancer cells is triggered by selective crosslinking and inhibition of receptor tyrosine kinases
Source: Commun Biol. 2019 Jun 21;2:231. doi: 10.1038/s42003-019-0484-5 (PMC6588694; doi:10.1038/s42003-019-0484-5)
Supplement: Supplementary file 4 — Reporting Summary [file 42003_2019_484_MOESM4_ESM.pdf]

nature research

Corresponding author(s): Gen He

Last updated by author(s): May 14, 2019

Reporting Summary

Nature Research wishes to improve the reproducibility of the work that we publish. This form provides structure for consistency and transparency in reporting. For further information on Nature Research policies, see Authors & Referees and the Editorial Policy Checklist.

Statistics

For all statistical analyses, confirm that the following items are present in the figure legend, table legend, main text, or Methods section.

n/a	Confirmed	
☐	☒	The exact sample size (*n*) for each experimental group/condition, given as a discrete number and unit of measurement
☐	☒	A statement on whether measurements were taken from distinct samples or whether the same sample was measured repeatedly
☐	☒	The statistical test(s) used AND whether they are one- or two-sided<br>*Only common tests should be described solely by name; describe more complex techniques in the Methods section.*
☒	☐	A description of all covariates tested
☐	☒	A description of any assumptions or corrections, such as tests of normality and adjustment for multiple comparisons
☐	☒	A full description of the statistical parameters including central tendency (e.g. means) or other basic estimates (e.g. regression coefficient) AND variation (e.g. standard deviation) or associated estimates of uncertainty (e.g. confidence intervals)
☒	☐	For null hypothesis testing, the test statistic (e.g. F, t, r) with confidence intervals, effect sizes, degrees of freedom and P value noted<br>*Give P values as exact values whenever suitable.*
☒	☐	For Bayesian analysis, information on the choice of priors and Markov chain Monte Carlo settings
☐	☐	For hierarchical and complex designs, identification of the appropriate level for tests and full reporting of outcomes
☒	☐	Estimates of effect sizes (e.g. Cohen's *d*, Pearson's *r*), indicating how they were calculated

Our web collection on statistics for biologists contains articles on many of the points above.

Software and code

Policy information about availability of computer code

Data collection	SPSS 19.0
Data analysis	All statistical evaluations were analyzed by one-way analysis of variance (ANOVA) followed by Tukey's HSD post hoc test using the software SPSS 19.0. Tukey's HSD post hoc test was used to examine treatment differences among the interactions. When the interaction was significant, the results were further analyzed using one-way ANOVA and Turkey's HSD post hoc test. In case unequal variance was determined by Levene's test, the data were square root-transformed before statistical analysis. A value of P < 0.05 was considered statistically significant. Each value is expressed as means ± S.E.M.

For manuscripts utilizing custom algorithms or software that are central to the research but not yet described in published literature, software must be made available to editors/reviewers. We strongly encourage code deposition in a community repository (e.g. GitHub). See the Nature Research guidelines for submitting code & software for further information.

Data

Policy information about availability of data

All manuscripts must include a data availability statement. This statement should provide the following information, where applicable:

- Accession codes, unique identifiers, or web links for publicly available datasets
- A list of figures that have associated raw data
- A description of any restrictions on data availability

All data generated or analysed during this study are included in this published article (and its supplementary information files).

Field-specific reporting

Please select the one below that is the best fit for your research. If you are not sure, read the appropriate sections before making your selection.

☒ Life sciences ☐ Behavioural & social sciences ☐ Ecological, evolutionary & environmental sciences

For a reference copy of the document with all sections, see nature.com/documents/nr-reporting-summary-flat.pdf

Life sciences study design

All studies must disclose on these points even when the disclosure is negative.

Sample size	No sample-size calculations were performed.
Data exclusions	No data were excluded.
Replication	All experiments were reproduced to reliably support conclusions stated in the manuscript.
Randomization	Cells were randomly divided into experimental groups.
Blinding	We preformed an unbiased analysis to all datasets.

Reporting for specific materials, systems and methods

We require information from authors about some types of materials, experimental systems and methods used in many studies. Here, indicate whether each material, system or method listed is relevant to your study. If you are not sure if a list item applies to your research, read the appropriate section before selecting a response.

Materials & experimental systems

n/a	Involved in the study	
☐	☒	Antibodies
☐	☒	Eukaryotic cell lines
☒	☐	Palaeontology
☒	☐	Animals and other organisms
☒	☐	Human research participants
☒	☐	Clinical data

Methods

n/a	Involved in the study	
☒	☐	ChIP-seq
☐	☒	Flow cytometry
☒	☐	MRI-based neuroimaging

Antibodies

Antibodies used	BCL2 (CST, # 4223), phospho-BAD (Ser136) (CST, # 4366), BAD (CST, # 9239), CASP9 (CST, # 9502), CASP3 (CST, # 9665), CASP8 (CST, # 9746), BECN1 (CST, # 3738), ATG12 (CST, # 4180), phospho-INSR (Tyr1150/1151) (CST, # 3024), INSR (CST, # 3020), IGFLR1 (CST, # 3027), EGFR (CST, # 2232), MET (Santa Cruz, # 8057), phospho-TGFBR1 (Thermo, # PA5-40298), TGFBR1 (Santa Cruz, # 518018), TNFR1 (Santa Cruz, # 8436), DR4 (Santa Cruz, # 8411), phospho-Tyr (PY20) (Thermo, # MA12439), phospho-AKT (Thr308) (CST, # 13038), phospho-AKT (Ser473) (CST, # 3787), AKT (CST, # 9272), phospho-MAPK1/2 (Thr202/Tyr204) (CST, # 4370), MAPK1/2 (CST, # 9107), phospho-RPS6KB1 (Thr389) (CST, # 9205), RPS6KB1 (CST, # 9202), phospho-FOXO1 (Ser256) (CST, # 9461), FOXO1 (CST, # 2880), phospho-FOXO3A (Ser253) (CST, # 9466), FOXO3A (CST, # 2497), phospho-PRKAA1 (Thr172) (CST, # 50081), PRKAA1 (CST, # 5831), phospho-EIF2A (Ser51) (CST, # 3597), EIF2A (CST, # 9722), TP53 (CST, # 2527), phospho-IKK[illegible]/[illegible] (Ser176/180) (CST, # 2679), CHUK (CST, # 11930), IKBKB (CST, # 8943), HA-tag (CST, # 3724), [illegible]-TUBULIN (CST, # 2146).
Validation	All antibodies were used in the system under study (assay and species) according to the profile of manufacturer.

Eukaryotic cell lines

Policy information about cell lines

Cell line source(s)	the Type Culture Collection of the Chinese Academy of Sciences, Shanghai, China.
Authentication	All cell lines authenticated by STR method by the Type Culture Collection of the Chinese Academy of Sciences, Shanghai, China.
Mycoplasma contamination	All cell lines were tested negative for mycoplasma contamination by the Type Culture Collection of the Chinese Academy of Sciences, Shanghai, China.

Commonly misidentified lines (See ICLAC register)	None of the used cell lines is listed in ICLAC database.

Flow Cytometry

Plots

Confirm that:

☒ The axis labels state the marker and fluorochrome used (e.g. CD4-FITC).

☒ The axis scales are clearly visible. Include numbers along axes only for bottom left plot of group (a 'group' is an analysis of identical markers).

☒ All plots are contour plots with outliers or pseudocolor plots.

☒ A numerical value for number of cells or percentage (with statistics) is provided.

Methodology

Sample preparation	Sample preparation listed in Methods
Instrument	BD Accuri™ C6 flow cytometer
Software	CFlow Plus
Cell population abundance	Cell sorting not employed
Gating strategy	Cells in the Q1-LL: AV-/PI- quadrant: live cells; Q1-LR: AV+/PI- quadrant: early apoptotic cells; Q1-UR: AV+/PI+ quadrant: late apoptotic cells and Q1-UL: AV-/PI+ quadrant: necrotic cells.

☒ Tick this box to confirm that a figure exemplifying the gating strategy is provided in the Supplementary Information.
